# The Uterine Cancer Committee of the British Medical Association

**Published:** 1909-05-15

**Authors:** 


### THE UTERINE CANCER COMMITTEE OF THE BRITISH MEDICAL ASSOCIATION.

### AN APPEAL TO MEDICAL PRACTITIONERS FOR THE EARLIER RECOGNITION OF UTERINE CANCER.

By the courtesy of the Editor of the *British Medical Journal*, we are enabled to publish herewith an abstract of the appeal to which reference is made in our leading article in the present issue. The committee calls the attention of all medical practitioners to the necessity of emphasising the curability by operation of uterine cancer in its early stages. The adoption of a more extensive operation by the abdominal route has made it possible to deal successfully with cases hitherto regarded as inoperable, and to remove more of the pelvic cellular tissue as well as a portion of the vaginal walls; it is in these situations that recurrence is prone to develop. Many patients now present themselves for examination and treatment when the disease is considerably advanced.

Special attention is directed to the facts that cancer of the uterus is at first a local disease; it is often curable; operation is the only satisfactory method of treatment; the earlier the disease is recognised the more hopeful are the prospects of treatment; the risk of operation in early cases is slight, and the chance of permanent cure is good; the recognition of early cancer is not usually difficult. A medical practitioner who fails to make a physical examination of a patient exhibiting any of the symptoms of uterine cancer incurs grave responsibility, since treatment of symptoms without physical examination is unjustifiable. In doubtful cases a diagnosis must and can be made in a few days. To examine, to diagnose, and then to treat, should be the rule in all cases.

The committee then give a *résumé* of the symptoms, methods of examination, varieties and microscopical appearances of cancer of the uterus. They emphasise the fact that as the majority of cases occur between the fortieth and fiftieth year, the symptoms are too often regarded by the patient as due to "change of life." The medical attendant should not accept this assumption until he is satisfied that cancer does not exist. Bleeding, however slight, occurring after the menopause, should give rise to suspicion that cancer is present.

If a patient with any of the above symptoms comes for advice, a careful visual and bi-manual examination must be made before any treatment is recommended. Should a patient refuse to be examined—and this is exceptional when the situation is explained—the medical attendant should decline any further responsibility, and no treatment should be advised. The examination should be made, even if bleeding is present, as valuable time may be lost by postponement until the hæmorrhage has ceased. It is most important to observe rigid aseptic precautions in all manipulations. In the examination, the condition of the vaginal portion of the cervix and of the cervical canal should be carefully noted. The detection of high-lying cervical cancers, and cancers of the body of the uterus, is only possible after curettage or digital exploration.

The signs common to the early stages of cancer of the cervix uteri are:—

(1) The definite occurrence of new growth on the surface of the vaginal portion of the cervix, in the lining of the cervical canal, or in the substance of the cervix.

(2) Friability.

(3) Bleeding on manipulation.

(1) The definite occurrence of new growth on the portio vaginalis or in the cervical canal cannot fail to arouse suspicion. When, however, thickening of one lip of the cervix exists, the nature of the growth is difficult to determine if the mucous covering be still intact.

(2) Friability is a sign of the greatest importance, and may be tested by the finger-nail, curette, sound, or an ordinary long probe.

(3) The occurrence of free bleeding after the slightest manipulation is, when combined with friability, a valuable diagnostic aid.

The committee classifies the forms of uterine cancer into:—

(A) Vaginal Portion of the Cervix.

(1) Infiltrating type.
(2) Papillomatous or polypoid type.
(3) Superficial flattened type.

(B) Cervical Canal.

(1) Superficial type.
(2) Infiltrating type.

They draw especial attention to the fact that probably the majority of cancer cases which are overlooked are examples of disease affecting the lining of the cervical canal or the tissues of the wall of the cervix. Cancer beginning in the cervical canal is not difficult to detect where the os uteri is dilated, as in many multiparæ. The finger passed into the cervical canal feels irregular elevations or nodules from which portions may be removed. Free hæmorrhage follows this manipulation. Difficulty arises where the os uteri is not dilated and the disease is hidden. A sound carefully passed into the cervical canal may give the impression of impinging on an irregular nodular surface, or friable tissue may be removed by the curette. Free hæmorrhage following such manipulations is a suspicious sign. Thickening and hardening of the cervix may be detected by a rectal examination, which is most helpful in detecting cancerous nodules in the cervical walls, and should always be made in such cases.

With regard to microscopical investigation, they formulate the excellent rule that in doubtful cases, if there be a suspicious hard nodule, or erosion, or ulcer on the external os uteri, a piece including a boundary of healthy tissue should be excised in the manner fully described in their appeal. "If the expert's report is favourable the patient will be reassured, if unfavourable immediate operation is imperative." As a summary they give the following axioms:—

(1) Attend to all symptoms suspicious of cancer, and instruct the patient on their importance;

(2) Examine immediately all cases of bleeding or abnormal discharge;

(3) Make a definite diagnosis, and do not wait for the disease to develop;

(4) Urge immediate operation if the diagnosis is established.

The practitioner who diagnoses cancer in an early stage, when operation offers a probability of cure, renders a service to his patient as great as that rendered by the operator.

---

Following the appeal to medical men appears—

A Short Appeal to Midwives and Nurses, which runs as follows:—

Cancer of the womb is a very common and fatal disease in women, but *it can be cured by operation when it is recognised early.* A woman sometimes tells a nurse or midwife her ailments before she speaks to a doctor, and the nurse or midwife has then an opportunity of aiding our crusade against this terrible disease. *Cancer may occur at any age, and in a woman who looks quite well and who may have no pain, no wasting, no foul discharge, and no profuse bleeding.*

To wait for pain, wasting, foul discharge, or profuse bleeding is to throw away the chance of successful treatment.

The early signs of cancer of the womb are:—

(1) *Bleeding*, which occurs after the change of life;

(2) *Bleeding* after sexual intercourse, or after a vaginal douche;

(3) *Bleeding*, slight or abundant, even in young women, if occurring between the usual monthly periods, and especially when accompanied by a bad-smelling or watery blood-tinged discharge;

(4) *Thin watery discharge* occurring at any age.

The nurse or midwife who is told by a patient that she has any of these symptoms should insist upon her seeing a medical practitioner in order that an examination may be made without delay. By doing so she will often help to save a valuable life, and will bring credit to herself and to her calling.

---

[The Committee's Appeal is printed in full in the current issue of the *British Medical Journal* (May 15, 1909).]

